# Cerebral and splanchnic near-infrared spectroscopic dataset in premature newborns receiving packed red blood cell transfusion

**DOI:** 10.1016/j.dib.2022.108824

**Published:** 2022-12-16

**Authors:** Kiran Kumar Balegar V, Madhuka Jayawardhana, Philip de Chazal, Ralph Kay Heinrich Nanan

**Affiliations:** a Department of Neonatology, Nepean Hospital, Sydney Medical School Nepean, The University of Sydney, NSW, Australia; b School of Electrical Engineering and the Charles Perkins Center, The University of Sydney, Australia; c School of Biomedical Engineering and the Charles Perkins Center, The University of Sydney, Australia; d The University of Sydney, Charles Perkins Center Nepean, Australia

**Keywords:** Preterm, Neonate, Tissue oxygenation, Splanchnic-cerebral oxygenation ratio, Packed Red Blood Cell Transfusion

## Abstract

This article presents the near-infrared spectroscopy (NIRS) dataset of cerebral (StO_2_c) and splanchnic (StO_2_s) oxygenation in 29 stable premature infants admitted to a tertiary neonatal intensive care unit who received elective packed red blood cell transfusion (PRBCT) to treat anemia of prematurity.

StO_2_c and StO_2_s data were prospectively recorded continuously from at least 4 hours before the beginning of PRBCT until 24 hours after its completion, using a 4-wavelength near-infrared spectroscopy (NIRS) monitor (FORE-SIGHT® absolute cerebral oximeter, CASMED, Branford, Connecticut, 06405 USA). StO_2_ data were downloaded as an analog output at a sampling rate of 1000Hz and aligned along the time axis in LabChart reader format (.adicht files) using a PowerLab data acquisition system [1] (PowerLab®, ADInstruments, Sydney, Australia). The .adicht files were then converted into .mat file format using a Python script (Python^TM^ version 3.7.3 [2]) and resampled at 1Hz for faster processing.

Data that could not be physiologically explained (e.g., the absence of variability, [3] a 30% step change in StO_2_ between two subsequent data points for StO_2_[4]), as well as the data during the period of ‘cares’ were presumed to be artefactual and were replaced with ‘NaN’ or ‘Not a Number’ which is recognised by Matlab [5] (MATLAB 9.3, The MathWorks, Inc., Massachusetts, United States) and ignored for all subsequent processing while maintaining the correct time point of the StO_2_ signals. The data were then exported into Microsoft Excel format. The splanchnic cerebral oxygenation ratio (SCOR) was calculated as the ratio of StO_2_s/StO_2_c. A 4-hour mean pre-transfusion values (StO_2_s 0, StO_2_c 0, SCOR 0) and post-transfusion hourly mean values (1-28) were determined. Secondary data were derived from a Mixed Models for Repeated Measures (MMRM) analysis with the time point fitted as a fixed effect and the infant fitted as a random effect. The MMRM was used to perform paired comparisons between pre-transfusion and each of the post-baseline values.

This article only provides the NIRS data. The secondary data and demography can be found in the article “Splanchnic-Cerebral Oxygenation Ratio associated with Packed Red Blood Cell Transfusion in preterm infants”, published in Transfusion Medicine. [6] The data will be of use to researchers in neonatology, transfusion medicine, and physiology to understand changes in cerebral and splanchnic oxygenation associated with PRBCT. Data collection, processing, and analysis can be remodelled in larger multicentric randomised controlled studies to evaluate the effect of transfusion and feeding on transfusion-associated necrotising enterocolitis. The data are also helpful to explore the autoregulatory behaviour of the brain and gut when the oxygen content of blood is increased by administering PRBCT.

**Specifications Table**

**Table:** 

Subject	Health and medical sciences
Specific subject area	Neonatology, transfusion medicine
Type of data	Table
How the data were acquired	Cerebral (StO_2_c) and splanchnic (StO_2_s) oxygenation data were recorded using a 4-wavelength near-infrared spectroscopy (NIRS) monitor (FORE-SIGHT® absolute cerebral oximeter, CASMED, Branford, Connecticut, 06405 USA). Data were aligned along the time axis and downloaded into a computer using a PowerLab data acquisition system [1] (PowerLab®, ADInstruments, Sydney, Australia)**.** Artefactual data were filtered out using the software Matlab [5] (MATLAB 9.3, The MathWorks, Inc., Massachusetts, United States).
Data format	Raw and filtered
Description of data collection	NIRS data were collected using a PowerLab data acquisition system, [1] filtered out using Matlab [5] software and exported to Microsoft Excel.
Data source location	• Institution: Nepean Hospital • City/Town/Region: Kingswood, New South Wales • Country: Australia • Latitude and longitude (and GPS coordinates) for collected data: 33.7593° S, 150.7137° E
Data accessibility	Freely available using the link https://data.mendeley.com/datasets/68g278cx8t/1
Related research article	The article is published in Transfusion Medicine as follows: Kiran Kumar Balegar V, Jayawardhana M, de Chazal P, Nanan RKH. Splanchnic-cerebral oxygenation ratio associated with packed red blood cell transfusion in preterm infants. Transfus Med. 2022 Oct 12. doi:10.1111/tme.12919. Epub ahead of print. PMID:36222235.

Value of the Data

• The data would benefit researchers in neonatology, transfusion medicine, and physiology to understand the changes in cerebral and splanchnic tissue oxygenation before, during, and after PRBCT.
• The data are useful to explore the autoregulatory behaviour of the brain and gut when the oxygen content of blood is increased by transfusing PRBCT.
• The data may also be relevant in the context of transfusion-associated necrotising enterocolitis (TANEC).
• Data collection, processing, and analysis can be remodelled in larger multicentric studies to evaluate the association of PRBCT with cerebral and splanchnic tissue oxygenation. It can also be remodelled in randomised controlled studies to assess the effect of feeding on TANEC.

## 1 Objective

The data set was generated with a view to providing a detailed description of cerebral and splanchnic StO_2_. The aim of the original study [6] was to examine the changes in StO_2_s, StO_2_c, and SCOR values associated with PRBCT. Accordingly, these values were monitored before, during and after PRBCT. Change in trend was evaluated in relation to PRBCT. Summary data provided in the published article [6] gives a trend of hourly mean values of StO_2_ over 28 hours. Hourly mean values were chosen for pragmatic reasons. However, it may be of further interest to researchers to analyse the trends over shorter data-averaging periods. Data averaging of 5 minutes have been recommended to overcome errors involved in splanchnic StO_2_ (and SCOR) measurements due to fluctuations in splanchnic oxygenation readings owing to peristaltic changes in the sampled splanchnic tissue. [7], [8], [9]

## 2 Data Description

The filtered NIRS raw data of 29 premature infants who received PRBCT has been provided in the repository as Microsoft Excel and accessible on the Mendeley data set using the link https://data.mendeley.com/datasets/68g278cx8t/1. NIRS data computed using the raw data are presented in Table 1. Mean (95% CI) values of cerebral and splanchnic StO_2_ and SCOR during the 4 hours before the commencement of PRBCT and hourly mean (95% CI) values thereafter for the next 28 hours are presented.

**Table 1:** Cerebral and splanchnic oxygen kinetics in association with transfusion (N=29).

	Hours	Splanchnic StO_2_, Mean (95% CI)	Cerebral StO_2_, Mean (95% CI)	SCOR, Mean (95% CI)
Pre-PRBCT	0	83.4 (81.0 to 85.8)	71.5 (69.5 to 73.4)	1.17 (1.13 to 1.21)
PRBCT	1	82.0 (79.6 to 84.4)	71.4 (69.4 to 73.4)	1.15 (1.12 to 1.19)
	2	83.6 (81.2 to 85.9)	72.9 (70.9 to 74.9)	1.15 (1.11 to 1.19)
	3	85.4 (83.0 to 87.8)	73.1 (71.1 to 75.1)	1.18 (1.14 to 1.21)
	4	83.0 (80.6 to 85.4)	74.4 (72.4 to 76.4)	1.12 (1.08 to 1.16)
Post-PRBCT	5	83.8 (81.4 to 86.2)	75.6 (73.6 to 77.6)	1.11 (1.07 to 1.15)
	6	83.1 (80.7 to 85.5)	74.5 (72.5 to 76.5)	1.12 (1.08 to 1.15)
	7	84.1 (81.7 to 86.5)	76.3 (74.4 to 78.3)	1.10 (1.07 to 1.14)
	8	83.6 (81.2 to 86.0)	76.0 (74.0 to 78.0)	1.10 (1.06 to 1.14)
	9	83.2 (80.9 to 85.6)	75.2 (73.2 to 77.2)	1.11 (1.07 to 1.15)
	10	83.9 (81.5 to 86.2)	74.8 (72.8 to 76.8)	1.12 (1.08 to 1.16)
	11	84.5 (82.1 to 86.9)	74.6 (72.6 to 76.6)	1.14 (1.10 to 1.17)
	12	83.6 (81.2 to 86.0)	75.1 (73.1 to 77.1)	1.12 (1.08 to 1.15)
	13	83.8 (81.4 to 86.2)	75.3 (73.3 to 77.3)	1.12 (1.08 to 1.15)
	14	84.0 (81.6 to 86.4)	75.6 (73.6 to 77.6)	1.11 (1.08 to 1.15)
	15	82.0 (79.6 to 84.4)	74.9 (72.9 to 76.9)	1.10 (1.06 to 1.14)
	16	82.3 (79.9 to 84.7)	74.7 (72.7 to 76.7)	1.11 (1.07 to 1.14)
	17	82.6 (80.2 to 85.0)	75.2 (73.3 to 77.2)	1.10 (1.06 to 1.14)
	18	82.5 (80.1 to 85.0)	74.6 (72.6 to 76.6)	1.11 (1.07 to 1.15)
	19	81.5 (79.1 to 84.0)	74.5 (72.5 to 76.6)	1.10 (1.06 to 1.14)
	20	83.1 (80.6 to 85.5)	75.0 (73.0 to 77.1)	1.11 (1.07 to 1.15)
	21	83.8 (81.4 to 86.3)	73.8 (71.8 to 75.8)	1.14 (1.10 to 1.18)
	22	82.1 (79.7 to 84.5)	73.6 (71.6 to 75.6)	1.12 (1.08 to 1.16)
	23	83.3 (80.9 to 85.8)	74.0 (71.9 to 76.0)	1.13 (1.09 to 1.17)
	24	85.3 (82.9 to 87.8)	73.7 (71.7 to 75.7)	1.17 (1.13 to 1.21)
	25	83.5 (81.0 to 85.9)	74.5 (72.4 to 76.5)	1.13 (1.09 to 1.16)
	26	85.3 (82.9 to 87.7)	75.3 (73.3 to 77.4)	1.14 (1.10 to 1.18)
	27	83.8 (81.4 to 86.3)	75.3 (73.2 to 77.3)	1.12 (1.08 to 1.16)
	28	81.6 (79.2 to 84.1)	74.6 (72.6 to 76.6)	1.10 (1.06 to 1.14)

PRBCT, Packed Red Blood Cell Transfusion; StO_2_, tissue oxygenation; SCOR, Splanchnic-Cerebral oxygenation ratio

## 3 Experimental Design, Materials, and Methods

### 3.1 Data acquisition

A 4-wavelength near-infrared spectroscopy (NIRS) monitor (FORE-SIGHT® absolute cerebral oximeter, CASMED, Branford, Connecticut, 06405 USA) was used to perform tissue oxygen measurements.

Eligible participants included: gestation<32 weeks; birth weight <1500 g; postmenstrual age <37 weeks; tolerating enteral feed volume at least 120 ml/kg/day; hemodynamically stable and receiving elective PRBCT to treat anaemia of prematurity. Infants with necrotising enterocolitis (current or previous); feed intolerance (defined as the treating clinical team's decision to withhold feeds/withhold grading up of feeds for at least 12 h); sepsis (defined as the treating clinical team's decision to commence antibiotics); PRBCT in the previous 72 h; patent ductus arteriosus or its treatment in the previous 72 h and/or congenital gastrointestinal, complex cardiac/lethal anomalies were excluded. The demography of the enrolled babies is shown in Table 1 of the article. [6]

Once a clinical decision was made to provide PRBCT in an eligible infant, a non-adhesive neonatal sensor was placed over the temporal region of the head (either right or left) and kept in place using continuous positive airway pressure (CPAP) hat (if they needed CPAP) or a non-latex self-adherent wrap (Coban^TM^ NL, 3M Deutschland GmbH Health Care Business, Germany) if they did not need CPAP, to obtain StO_2_c. In our experience, non-adhesive sensors were better than adhesive sensors for cerebral monitoring. They are not only easy to be kept in place using a CPAP hat but also avoid trauma from lifting the sensor in neonates with an abundance of scalp hair. Splanchnic oxygenation (StO_2_s) was monitored by placing a neonatal sensor on the lower quadrant of the abdomen just below the umbilicus [3,10], held in place either by a nappy or using an adhesive sensor. PRBCT was initiated after obtaining at least 4 hours of recording of StO_2_c and StO_2_s. All infants received 15 mL/Kg PRBCT transfused over 4 hours period. The recording was obtained continuously during the 4 hours of PRBCT and for 24 hours after its completion. As there are no normative values for StO_2_, the alarms were silenced. Proper skin contact with sensors and appropriate recordings were ensured by the bedside nurse through recordings obtained every half-hourly. Arterial oxygen saturation (SpO_2_) was monitored using Masimo Radical-7 monitor. Skin integrity was closely monitored by lifting the sensors and inspecting the skin every 6 hours at the time of ‘cares’ (handling of neonates for a nappy change, eye care, change of posture). The same position of the sensor was maintained throughout the study.

**Fig 1:**
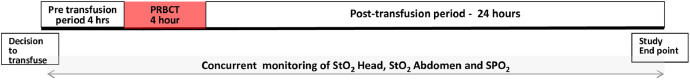
Outline of the study procedure.

Oxygenation data were downloaded in real-time from the NIRS monitor to a laptop as an analog output at a sampling rate of 1000Hz and aligned along the time axis in LabChart reader format (.adicht files) using a PowerLab data acquisition system [1] (PowerLab®, ADInstruments, Sydney, Australia)**.** The start and end times of the study, as well as various events, namely transfusion, feeds, and cares, were annotated in real-time in the PowerLab system. As a backup, the timings of these events were also recorded on a paper copy. Thus, each baby had a single, long .adicht file of at least 32 hours, with all the events embedded in it. The data was stored in the research laptop and backup drives. A total of 29 infants were enrolled in the study. The study procedure has been outlined in Fig. 1.

### 3.2 Data processing

This involved the following steps

#### 3.2.1 Reading the data files

By default, the acquisition system saves the data in LabChart reader format (.adicht files). These data were anonymised and exported in the Matlab standard data file format (.mat) using the LabChart reader. However, the .mat files produced in this export process were unreadable by Matlab 8.4 due to the sheer size of the records (32 hours at 1000Hz sampling rate). Therefore, these .mat data files were converted to Matlab 9 .mat data files using a Python script as the resulting .mat could then be read into and processed using Matlab 9.3. The data itself and the various events and parameters associated with the data, such as dates and times and sampling rates, were embedded in these data files in a specific structure. Matlab codes were written to extract the information in a convenient manner. To rectify this issue of lengthy data and to make the data processing fast and convenient, the data were resampled at 1Hz. Recordings of 29 infants out of the 30 were considered. One record was removed due to corrupted data that could not be read.

#### 3.2.2 Data alignment

After the data and the other relevant parameters and information were extracted from the data files, the next task was to match/align the start and end times of transfusion. First of all, the various events, such as the start-finish times of transfusion, cares, and feeding, embedded in the electronic files were manually verified against the paper records. The next task was to align the start of the transfusion for each infant. Although the transfusion was ordered over a period of 4 hours, in reality, the duration of the transfusion varied between babies by a few minutes. To circumvent this issue, the endpoint of transfusion was marked precisely 4 hours after the commencement of transfusion. The data collected for 24 hours after the endpoint of transfusion was taken as the post-transfusion period. The data collected for 4 hours before the commencement of transfusion was taken as the pre-transfusion period. Any record with excess data on either side of the study period was cut out. Short periods of missing data (possibly due to interruptions to sensor contact with the skin) were interpolated within these periods considering the trends before and after such an event.

#### 3.2.3 Data filtering and outlier correction

Several strategies of outlier correction based on literature and heuristics were adopted. Data that could not be physiologically explained (e.g., the absence of variability for 30 seconds or more, [3] a 30% step change in StO_2_ between two subsequent data points for StO_2_ [4]) were tagged as they were presumed to be artefactual. In addition, the data during the period of ‘Cares’ were also tagged because of the possibility of interruptions to sensor contact from removal for skin inspection and /or movement artifacts due to infant handling during this period. The tagged data were replaced with ‘NaN’ or ‘Not a Number’ which is recognised by Matlab [5] (MATLAB 9.3, The MathWorks, Inc., Massachusetts, United States) and ignored for all subsequent processing while maintaining the correct time point of the StO_2_ signals.

In addition, all the signals were carefully observed manually for any obvious discrepancies and outliers that were missed using the above automatic filtering steps, and any such occurrences were replaced by interpolated data. The final step in processing the StO_2_ data was to remove the noise in the data by applying a median filter of length of 300 seconds.

#### 3.2.4 Export of the filtered data

The processed data were exported into a Microsoft Excel file. Each baby had at least 32 hours of recording (5.61 hours pre-transfusion, 4 hours during transfusion, and 24 hours after the end of transfusion), with one StO_2_ value per second. SCOR was obtained as the ratio of the StO_2_s numerator versus the StO_2_c denominator. Data relevant to the linked article in the Journal Transfusion Medicine has been presented.

### 3.3 Data analysis

A mean (95% CI) value was obtained from the 4-hour pre-transfusion values to represent the baseline pre-transfusion StO_2_. Hourly mean (95% CI) values were obtained during and after PRBCT for the next 28 hours. The data are shown in Table 1. As seen in the table, cerebral StO_2_ values are more tightly distributed around the mean compared to splanchnic StO_2_ values. It is well known that splanchnic StO_2_ values are inherently associated with wider confidence intervals [7,^9^,11]. A physiological reason for this is the active peristalsis of the healthy bowel resulting in variable segments of the intestine being interrogated, as well as changing gas-fluid-fecal interfaces. [12]

The wider confidence intervals of splanchnic StO_2_ values can potentially compromise the reliability of splanchnic tissue oxygenation measurements. To circumvent this issue, Mintzer et al. [7] recommended utilising relatively shorter periods as a preferred data averaging interval and using these epochs to evaluate trends in splanchnic tissue oxygenation over longer periods. Thus, splanchnic tissue oxygenation is meaningful only if monitored continuously at high frequency over longer periods. Accordingly, our approach towards splanchnic oxygenation monitoring, data extraction, and analysis are superior to many other published studies. Unlike other studies [13], [14], [15], [16], where the sampling frequency varied from one per 6 sec to 1 minute, our sampling frequency was one per second. More frequent sampling is likely to pick up inherent changes compared to less frequent sampling. We performed continuous rather than episodic monitoring so as to not miss out on changes occurring at other times in the specified period of monitoring. Many studies, on the other hand, performed episodic measurements varying from spot measurement [13] to monitoring over 20 minutes. [14], [15], [16] Even though some studies performed longer duration of monitoring over 4 to 11 hours, [17,18] they employed longer data averaging period and used a single mean ± SD value over the entire period of 4-11 hours, which obviously obscures the variability in NIRS recordings during this period. We calculated mean ± SD every hour during and after PRBCT to determine how splanchnic oxygenation varied during this period. Therefore, we believe that our approach has more adequately captured splanchnic variability, and overcome caveats associated with monitoring of splanchnic oxygenation.

Secondary data were obtained using a Mixed Models for Repeated Measures (MMRM) analysis to perform paired comparisons between the pre-transfusion mean value and each of the post-transfusion hourly mean values, as described in the manuscript published in the journal “Transfusion Medicine” [6].

## Ethics Statements

The study protocol, including parental consent, was approved by the human research ethics committee, Nepean Blue Mountain Local Health Committee (Approval number: Study 12/67 - HREC/12/NEPEAN/148).

## CRediT Author Statement

**Kiran Kumar Balegar V:** Conceptualisation, Methodology, data acquisition, original draft preparation, editing and final manuscript; **Madhuka Jayawardhana:** Data curation, original draft preparation; **Philip de Chazal:** Data curation, Supervision, Reviewing and Editing; **Ralph Kay Heinrich Nanan:** Supervision, Reviewing and Editing.

## Declaration of Competing Interest

The authors declare that they have no known competing financial interests or personal relationships that could have appeared to influence the work reported in this paper.

## Data Availability

NIRA transfusion data set (Original data) (Mendeley Data).
